# Acetylcholine in the hippocampus: problems and achievements

**DOI:** 10.3389/fncir.2025.1491820

**Published:** 2025-04-30

**Authors:** Yulia V. Dobryakova, Alexey P. Bolshakov, Tinna Korotkova, Andrey V. Rozov

**Affiliations:** ^1^Laboratory of Neurophysiology of Learning, Institute of Higher Nervous Activity and Neurophysiology, Moscow, Russia; ^2^Laboratory of Molecular Neurobiology, Institute of Higher Nervous Activity and Neurophysiology, Moscow, Russia

**Keywords:** medial septum area, acetylcholine, hippocampus, cholinergic neurons, GABA

## Abstract

Cholinergic septohippocampal projections originating from the medial septal area (MSA) play a critical role in regulating attention, memory formation, stress responses, and synaptic plasticity. Cholinergic axons from the MSA extensively innervate all hippocampal regions, providing a structural basis for the simultaneous release of acetylcholine (ACh) across the entire hippocampus. However, this widespread release appears inconsistent with the specific functional roles that ACh is thought to serve during distinct behaviors. A key unresolved question is how the dynamics of ACh tissue concentrations determine its ability to activate different receptor types and coordinate individual synaptic pathways. Here, we highlight several debated issues, including the potential intrinsic source of ACh within the hippocampus – such as cholinergic interneurons – and the co-release of ACh with GABA. Furthermore, we discuss recent findings on *in vivo* ACh concentration dynamics, which present a new dilemma for understanding ACh signaling in the hippocampus: the contrast between “global” ACh release, driven by synchronous activation of MSA neurons, and “local” release, which may be influenced by yet unidentified factors.

## 1 Complexity of functional cholinergic control of the hippocampal formation

It has been well established that ACh in the brain is involved in the regulation of attention, memory formation, stress response, and pain processing in rodents (Leaderbrand et al., 2016; Mineur et al., 2022; Sullere et al., 2023). One of the brain structures where ACh plays an important role is the hippocampus. Presumably, the only source of hippocampal cholinergic innervation is the MSA which includes the medial septal nucleus and diagonal band of Broca. ACh levels in the hippocampus increase under various conditions, including during learning and memory formation (Hironaka et al., 2001), development of stress response (Imperato et al., 1991; Mitsushima et al., 2003), and motor activity that does not require spatial navigation, for instance, treadmill running (Dudar et al., 1979). Such a variety of different conditions under which ACh levels increase raises questions about the functions performed by this neuromodulator within the same structure in different behavioral conditions. It may be speculated that the functional specificity of ACh is determined by ACh release targeting hippocampal neuronal subpopulations that are involved in the generation of behaviorally driven network activity. However, cholinergic axons originating in the MSA cover all hippocampal regions, which seems to provide the structural basis for simultaneous ACh release along entire hippocampus. These global changes in ACh concentration appear to be inconsistent with the specific function that the ACh released during a particular behavior is intended to serve. For quite a long period of time volume transmission has been generally accepted as the main modality of ACh release, and this has been acknowledged in many comprehensive reviews summarizing aspects of cholinergic signaling in the hippocampus (Hasselmo, 2006; Heys et al., 2012; Teles-Grilo Ruivo and Mellor, 2013). Previous studies opposed “volume” and “synaptic” modes of transmission, emphasizing the significance of this distinction (Descarries et al., 1997; Vizi and Kiss, 1998; Zoli et al., 1999). However, recent studies on *in vivo* ACh dynamics and structure of cholinergic MSA terminals suggest that ACh transmission does not strictly adhere to a single mode but instead renders of a combination of them, as will be discussed below. The question of how ACh tissue concentration dynamics determines the ability of this neuromodulator to activate different types of receptors and coordinate individual synaptic pathways remains largely unanswered. We believe that recent data have revealed important details that offer an updated perspective on the functional consequences of ACh volume transmission.

## 2 Anatomical organization of septohippocampal connections

Functional and anatomical organization of cholinergic projections to the hippocampal formation has been thoroughly described in previous reviews (Heys et al., 2012; Teles-Grilo Ruivo and Mellor, 2013). Here, we will briefly recapitulate the major synaptic targets of septal projections to the hippocampus. In general, MSA projections are formed by glutamatergic, GABAergic, and cholinergic neurons (Desikan et al., 2018). GABAergic projections constitute roughly one third of MSA afferents that originate predominantly from parvalbumin-positive GABAergic neurons and terminate on inhibitory hippocampal neurons located in CA1 and DG (Unal et al., 2015); therefore, their activation can lead to disinhibition of these hippocampal areas (Salib et al., 2019). Glutamatergic fibers originating in the MSA preferentially project to interneurons in the CA1 and CA3 areas of the hippocampus (Huh et al., 2010; Müller and Remy, 2018).

The axons of cholinergic MSA neurons can be found in close proximity to cell bodies and dendrites of neurons throughout the hippocampus. The highest density of cholinergic axons was observed in the alveus, the stratum oriens, and the stratum lacunosum moleculare (Heys et al., 2012). Cholinergic MSA projections innervate all hippocampal areas and neuron types. However, effects of activating these projections are more complex compared to GABAergic and glutamatergic projections. First, ACh may exert both inhibitory and excitatory effects depending on the receptor subtypes located on the postsynaptic neuron. For instance, it was shown that activation of cholinergic projections to entorhinal cortex from the MSA elicits hyperpolarizing and depolarizing responses mediated by M1 and M2 muscarinic receptor subtypes, respectively (Desikan et al., 2018). While hippocampal principal neurons predominantly express M1 and M3 receptors, hippocampal interneurons also express M2 receptors (Zeisel et al., 2015; Langlieb et al., 2023), suggesting that ACh release from MSA fibers can simultaneously activate some neurons while inhibiting others.

A second, equally important aspect of cholinergic signaling is co-transmission of ACh with GABA (Granger et al., 2023; Saunders et al., 2015; Takács et al., 2018). The co-transmission implies that activation of cholinergic MSA neurons induces a complex postsynaptic response in hippocampal neurons combining ACh-and GABA-mediated responses. However, the extent of ACh and GABA co-transmission from all MSA cholinergic fibers remains debated. While cholinergic neurons express GABA-synthesizing enzymes (Gad1 and Gad2), *in situ* hybridization revealed the vesicular GABA transporter (vGAT, Slc32a1) in only about 25% of cholinergic MSA neurons (Granger et al., 2023). A single-cell RNAseq study comparing striatal and basal forebrain cholinergic neurons found that all examined MSA neurons expressed Gad1, Gad2, Slc32a1, and genes for GABA plasma membrane transporters (GAT1/Slc6a1, GAT3/Slc6a3) (Rumpler et al., 2023). These findings suggest that while MSA cholinergic neurons possess the molecular machinery for GABA and ACh co-transmission, the variability in vGAT expression indicates potential regulation of GABAergic identity by specific factors or stimuli.

Thus, cholinergic neurons of the MSA send widespread projections along the entire longitudinal axis of the hippocampus as well as to the entorhinal cortex. Other areas of the neocortex receive cholinergic projections from the nucleus basalis of Meynert (Chen et al., 2023). Intraseptal GABAergic, cholinergic and glutamatergic cells are mutually connected (Takeuchi et al., 2021) providing a structural basis for synchronization of septal neuronal activity and synchronous ACh release in both the hippocampus and entorhinal cortex. However, so far, the synchrony of ACh release in different parts of the hippocampus and entorhinal cortex as well as between the hippocampus and entorhinal cortex has not been studied. A recent study showed that ACh release may be, at least partly, coordinated between the medial prefrontal cortex and dorsal hippocampus suggesting synchrony between different cholinergic nuclei (Teles-Grilo Ruivo et al., 2017). This notion is supported by the finding that an increase in ACh concentration in the different neocortical areas occurs in a highly coordinated manner during behavioral load with desynchronization occurring during locomotion (Lohani et al., 2022).

## 3 Cholinergic interneurons in the hippocampus

As noted before the MSA is a major source of cholinergic innervation to the hippocampus. However, it remains unclear whether additional sources of ACh exist within the hippocampus itself. One highly debated potential source is hippocampal cholinergic interneurons. Initially, these interneurons were identified using immunostaining for choline acetyltransferase (ChAT) in hippocampal slices (Frotscher et al., 2000; Takács et al., 2018; Yi et al., 2015). However, subsequent studies using transgenic mice designed to express green fluorescent protein (GFP) in cholinergic neurons failed to confirm the presence of cholinergic cells in the hippocampus. While GFP expression colocalized with ChAT immunostaining in cholinergic neurons of the striatum and basal forebrain, no such colocalization was observed in the hippocampus (Gamage et al., 2023). This discrepancy was attributed to potential misexpression of GFP in the hippocampus, leading to the recommendation that these transgenic mice be used only to label cholinergic neurons in well-established cholinergic regions (Blusztajn and Rinnofner, 2016). Importantly, these findings did not definitively disprove the existence of cholinergic interneurons in the hippocampus.

Parallel single-cell RNA-sequencing studies have revealed that some hippocampal interneurons—specifically, those expressing vasoactive intestinal peptide (VIP) – also express ChAT mRNA, along with other cholinergic markers such as the vesicular acetylcholine transporter (VAChT, slc18a3) and the choline transporter (ChT1, slc5a7) (Langlieb et al., 2023; Zeisel et al., 2015, 2018). However, the mRNA levels of these genes in hippocampal interneurons were approximately 100 times lower than those in basal forebrain cholinergic neurons, which corresponds to a few transcripts per cell (Zeisel et al., 2018).

It is worth noting that GFP expression in transgenic mice was found not in arbitrary hippocampal interneurons but in VIP-positive interneurons (Yi et al., 2015) which coincides with the aforementioned data of single cell RNA-seq. The detection of low ChAT mRNA expression in VIP-positive hippocampal interneurons suggests that GFP expression in the hippocampi of transgenic mice may not be an artifact but instead reflects a biological reality in which ChAT immunostaining (and *in situ* hybridization) yields negative results despite the expression of cholinergic genes. It is likely that such low mRNA levels result in correspondingly low levels of ChAT protein, which may be insufficient to produce a detectable immunostaining signal comparable to that of MSA cholinergic neurons. This could explain the apparent absence of ChAT staining in hippocampal GFP-positive interneurons in transgenic mice.

These findings raise a question: Does the presence of extremely low levels of ChAT mRNA (along with VAChT and ChT1 mRNAs) qualify a neuron as cholinergic? By definition, a cholinergic neuron is one that releases acetylcholine. Yet, when ChAT protein is present at minimal levels, it is unclear whether the amount of ACh synthesized would be sufficient to form a distinct pool of ACh-containing vesicles, the release of which could have a functional impact. Therefore, although recent studies have advanced our knowledge on the hippocampal cholinergic interneurons, it remains difficult to draw definitive conclusions about their cholinergic identity or the functional significance of the low expression of cholinergic proteins in these neurons. Resolution of the mentioned problems requires development of very sensitive techniques that would allow to detect release of single vesicles with ACh from selective neuronal subpopulations.

The above findings also raise a question about mechanisms that provide extremely low expression of cholinergic transcripts in some VIP-positive hippocampal interneurons. It remains entirely unclear whether the observed mRNA reflects stably low expression of genes related to cholinergic transmission or whether their expression is transiently induced in these neurons by some stimuli, temporarily rendering them cholinergic. Presumably, this transient nature could account for the low expression levels of cholinergic genes. Additional thorough experiments are required to clarify factors that determine development of cholinergic identity in some VIP-positive hippocampal interneurons.

## 4 Specific features of cholinergic signaling

The key to understanding the mechanisms underlying cholinergic modulation may be hidden in the functional diversity of ACh receptors. Acetylcholine can bind to two types of receptors, ionotropic nicotinic and metabotropic muscarinic receptors. According to numerous single cell RNA-seq studies (Langlieb et al., 2023; Zeisel et al., 2015, 2018), both types of receptors are expressed predominantly by neurons. While nicotinic homomeric α7-containing receptors are expressed in all neuronal types, hippocampal and cortical GABAergic interneurons express the α2–α5 and β2–β4 subunits of nicotinic receptors which form cation channels with various subunit compositions (Jackson et al., 2024). M1, M3 and M5 muscarinic receptors are coupled to Gq/11 proteins which activate phospholipase C and inositol triphosphate signaling pathways. In contrast, M2 and M4 muscarinic receptors are coupled to Gi/o proteins and their activation reduces cAMP level and mediates inhibitory effects.

Activation of muscarinic M1 and M3 receptors typically induces plasma membrane depolarization and facilitation of action potential firing in both principal cells and interneurons (Buchanan et al., 2010; Cea-del Rio et al., 2010; Chiang et al., 2010; Dasari and Gulledge, 2011; Fisahn et al., 2002; McQuiston and Madison, 1999). However, in some cases, M1 receptors activation leads to neuronal hyperpolarization which is usually followed by slow depolarization (Dasari and Gulledge, 2011; Gulledge and Kawaguchi, 2007; McQuiston and Madison, 1999). These receptors are widely expressed in the overwhelming majority of excitatory hippocampal neurons (Levey et al., 1995). While M3 receptors are found in both excitatory and inhibitory neurons, M1 receptors show more restricted expression, being present in only a subset of inhibitory interneurons at mRNA levels 2–3 times lower than in excitatory neurons (Langlieb et al., 2023; Saunders et al., 2018; Zeisel et al., 2015).

M4 muscarinic receptors are expressed in a subset of excitatory neurons, though at substantially lower levels compared to the M1 and M3 subtypes (Buckley et al., 1988; Langlieb et al., 2023; Saunders et al., 2018; Vilaró et al., 1993; Zeisel et al., 2015). Within inhibitory hippocampal interneurons, M4 receptor expression is even more limited, appearing at very low levels in only a small proportion of these cells (Langlieb et al., 2023; Saunders et al., 2018; Zeisel et al., 2015). Similarly, M5 receptors demonstrate restricted expression patterns, with detectable mRNA primarily in CA1 pyramidal cells (Vilaró et al., 1990, 1993) and a minor subpopulation of inhibitory neurons (Langlieb et al., 2023; Saunders et al., 2018; Zeisel et al., 2015). The low mRNA abundance of M4 and M5 receptors makes definitive conclusions about their physiological roles challenging. Nevertheless, emerging evidence indicates that M4 receptor activation can influence hippocampal interneuron excitability (Bell et al., 2013), suggesting these sparsely expressed receptors may still participate in modulating circuit function.

M2 receptors, inhibiting cellular activity and neurotransmitter release, are expressed in subpopulations of somatostatin-positive and parvalbumin-positive interneurons (Langlieb et al., 2023; Zeisel et al., 2015). Presumably, the selective expression of M2 receptors in dendrite-targeting and perisomatically projecting interneurons may play a fine-tuning role in modulation of hippocampal network activity by mediating suppression of GABA release from a selected set of synapses (Teles-Grilo Ruivo and Mellor, 2013).

The affinity of muscarinic receptors to acetylcholine is about 30 nM (Jackson et al., 2024; Kellar et al., 1985). Depending on subunit composition, nicotinic receptors have an EC50 in the range from 20 to 70 uM (Papke et al., 2007). However, the ACh concentration measured by microdialysis *in vivo* under resting conditions is about 1–8 nM (Hartmann et al., 2008; Mark et al., 1996). The use of restrained stress model or learning tasks as stimuli to promote ACh release caused only a twofold increase in its concentration (Chang and Gold, 2003; Imperato et al., 1991; Mark et al., 1996; Mitsushima et al., 2003, 2008). There appears to be a contradiction between the reported affinity of muscarinic and nicotinic receptors and tissue ACh concentrations measured by microdialysis. This contradiction has been partially resolved in experiments where ACh concentration dynamics has been measured using genetically encoded sensors. It is worth mentioning that the Kd of the sensors GACh2.0 and GrabAch3.0 is in the micromolar range suggesting that local ACh concentration must reach these values to produce a measurable signal (Jing et al., 2018, 2020). Fluctuations in ACh concentration can be detected using these sensors under a variety of behavioral conditions, including active learning, treadmill running, and restraint stress (Jing et al., 2018; Mei et al., 2020; Zhang et al., 2021). Thus, ACh levels in close proximity to the release site can most likely reach supramicromolar concentrations sufficient for activation of both nicotinic and muscarinic receptors expressed on target neurons. The concentration of ACh subsequently drops due to radial diffusion and the activity of acetylcholine esterase (AChE) reaching the values of a few nanomoles, as measured by microdialysis.

The above considerations suggest that released ACh seems to form some domains in extracellular space where its concentration is high enough to activate low affinity nicotinic receptors. This revives the old “wired” vs. “volume” dilemma for cholinergic transmission in the hippocampus. It was largely resolved by Takács et al. (2018), who demonstrated that cholinergic terminals of MSA axons form synaptic structures resembling inhibitory GABAergic synapses. In these synapses, co-transmitted GABA acts on clustered postsynaptic GABA receptors and, presumably, clustered nicotinic ACh receptors. This synapse differs from classical GABAergic synapses in terms of the clearance mechanisms: GABA is rapidly removed by transporters (GAT1 and GAT3) on pre-and postsynaptic membranes and surrounding astrocytes, whereas ACh is metabolized by extracellular AChE to choline, which is taken up via the ChT1 transporter on cholinergic terminals. Therefore, for GABA, this synapse seems to be typical synapse where GABA diffusion is limited though enabling its spillover under certain conditions. In contrast, the distance of ACh diffusion in it depends on local AChE activity rather than transporter proximity suggesting that, in some cases, high frequency activity of cholinergic neurons may overcome the spatial restrictions of ACh spreading dictated by radial diffusion and AChE activity. For instance, activation of hippocampal interneurons by cholinergic projections from MSA requires high stimulation frequencies (Mamad et al., 2015), which are not typical for cholinergic neurons *in vivo* (Ma et al., 2020). However, given that the firing rate of cholinergic neurons is usually quite low (1–10 Hz), and despite the extensive innervation of the hippocampus by cholinergic fibers, the effect of ACh release at low frequencies may be local and limited to neurons located close to the site of release (Jing et al., 2018, 2020). Notably, data on the *in vivo* firing patterns of cholinergic neurons during behavior remain limited, though evidence suggests they can generate synchronous high-frequency bursts (>100 Hz) under certain behavioral conditions (Hangya et al., 2015). Therefore, we can assume that the intensity of cholinergic signaling is determined by presynaptic bouton density, presynaptic firing rate, and local activity of AChE while the nature of the effect at the cellular level will depend on the type of ACh receptors involved.

Thus, the activation of MSA neurons, driven by intrinsic MSA connectivity, leads to the synchronization of these neurons and the simultaneous release of acetylcholine (ACh) and GABA across the entire hippocampus. This creates the impression that, despite synaptic ACh release, its global and synchronous release – followed by spillover – may eliminate any local ACh effects. However, two-photon imaging of ACh dynamics in the neocortex during treadmill running revealed that, although ACh release is globally synchronized across the neocortex with some heterogeneity between distantly located cortical areas (Lohani et al., 2022), it exhibits spatial heterogeneity at the level of neuronal ensembles (Jing et al., 2020). Specifically, ACh levels in regions separated by approximately 100 μm can display distinct dynamics suggesting that global changes are subject to local adjustments, leading to spatially heterogeneous ACh release. This raises questions about the factors governing these local adjustments and factors determining “global” vs. “local” ACh dynamics. While definitive data are lacking, the existing evidence points to several plausible mechanisms.

First, GABA co-released with ACh may contribute to spatial heterogeneity. Activation of GABA_B_ receptors has been shown to suppress ACh release (Jing et al., 2020). It is possible that the interplay between GABA released by local interneurons and cholinergic terminals creates a spatial pattern of GABA distribution, selectively suppressing some cholinergic terminals while leaving others unaffected. Another candidate for local ACh regulation is endocannabinoid system. MSA cholinergic neurons express cannabinoid receptors (Rumpler et al., 2023), and their activation can suppress ACh release (Gifford et al., 1997). Third, local hippocampal cholinergic interneurons may further shape spatial heterogeneity by releasing additional ACh during their activity. Fourth, serotonin may also either suppress or enhance ACh release, depending on conditions (Vizi and Kiss, 1998). Fifth, modulation of AChE activity could also contribute to this heterogeneity. For instance, beta-amyloid has been shown to increase AChE activity, leading to reduced ACh levels (Zueva et al., 2023) suggesting that local release or accumulation of endogenous amyloid could generate spatial heterogeneity of ACh level. Finally, heterogeneity in ACh release may result from suppression of intraseptal connectivity leading to desynchronization of MSA neurons and spatially heterogeneous ACh release in the hippocampus due to complex topical organization of MSA projections to the hippocampus (Dutar et al., 1995).

Recent findings have highlighted a significant relationship between ACh release in the hippocampus and various hippocampal activities. It was shown *in vivo* that ACh concentration greatly decreases during sharp-wave ripple (SWRs) while during theta oscillations the level of ACh increases (Zhang et al., 2021). The role of ACh in the transition between different oscillatory modalities was further confirmed by experiments where genetic activation of cholinergic MSA projections suppressed SWRs (Jarzebowski et al., 2021; Ma et al., 2020; Vandecasteele et al., 2014; Zhang et al., 2021, 2024). However, it was also shown that suppression of SWRs during MSA activation may be a result of GABA release from cholinergic fibers but not ACh release (Takács et al., 2018). Nevertheless, ACh still remains important driver of processes associated with gamma and theta oscillations. For example, it was shown that high ACh level during gamma oscillations leads to a decrease in oxytocin level in the hippocampus. Although, as it turned out, the connection between the release of ACh and oxytocin strongly depends on the state of the brain (i.e., REM sleep, non-REM sleep, wakefulness) (Zhang et al., 2024).

Thus, recent studies suggest that cholinergic signaling in the hippocampus has several important features: (1) activity of MSA cholinergic neurons seems to result in global ACh changes, presumably, associated with also global GABA co-transmission; (2) these global changes undergo local adjustments resulting in spatially heterogeneous ACh release; (3) the factors that may determine both global and local dynamics of ACh concentration have yet to be identified in future studies; and (4) it is currently unclear what functional role spatially heterogeneous ACh release plays in the hippocampus and future studies may help to unravel them.
